# Children of more empowered women are less likely to be left without vaccination in low- and middle-income countries: A global analysis of 50 DHS surveys

**DOI:** 10.7189/jogh.12.04022

**Published:** 2022-03-26

**Authors:** Andrea Wendt, Thiago M Santos, Bianca O Cata-Preta, Janaína C Costa, Tewodaj Mengistu, Daniel R Hogan, Cesar G Victora, Aluísio J D Barros

**Affiliations:** 1 International Center for Equity in Health, Federal University of Pelotas, Pelotas, Brazil; 2 Postgraduate Program in Epidemiology, Federal University of Pelotas, Pelotas, Brazil; 3 Gavi, the Vaccine Alliance, Geneva, Switzerland

## Abstract

**Background:**

To help provide a global understanding of the role of gender-related barriers to vaccination, we have used a broad measure of women’s empowerment and explored its association with the prevalence of zero-dose children aged 12-23 months across many low- and middle-income countries, using data from standardized national household surveys.

**Methods:**

We used data from Demographic and Health Surveys (DHS) of 50 countries with information on both women’s empowerment and child immunisation. Zero-dose was operationally defined as the proportion of children who failed to receive any doses of the diphtheria, pertussis, and tetanus containing vaccines (DPT). We measured women’s empowerment using the SWPER Global, an individual-level indicator estimated for women aged 15-49 years who are married or in union and with three domains: social independence, decision-making and attitude towards violence. We estimated two summary measures of inequality, the slope index of inequality (SII) and the concentration index (CIX). Results were presented for individual and pooled countries.

**Results:**

In the country-level (ecological) analyses we found that the higher the proportion of women with high empowerment, the lower the zero-dose prevalence. In the individual level analyses, overall, children with highly-empowered mothers presented lower prevalence of zero-dose than those with less-empowered mothers. The social independence domain presented more consistent associations with zero-dose. In 42 countries, the lowest zero-dose prevalence was found in the high empowerment groups, with the slope index of inequality showing significant results in 28 countries. When we pooled all countries using a multilevel Poisson model, children from mothers in the low and medium levels of the social independence domain had respectively 3.3 (95% confidence interval (CI) = 2.3, 4.7) and 1.8 (95% CI = 1.5, 2.1) times higher prevalence of zero-dose compared to those in the high level.

**Conclusions:**

Our country-level and individual-level analyses support the importance of women’s empowerment for child vaccination, especially in countries with weaker routine immunisation programs.

Despite impressive progress in introducing new vaccines in low- and middle-income countries (LMICs) in the past decade, there are approximately 13 million ‘zero-dose’ children year after year. These are children who did not receive the first dose of a diphtheria, pertussis, and tetanus (DPT) containing vaccine – a marker for access to routine immunisation and a proxy for children who received no doses of any basic vaccine through routine health systems [1]. The COVID-19 pandemic has further exacerbated the situation, pushing up the number to an estimated 17 million in 2020 [2]. As immunisation plays an essential role in child health by reducing deaths and disabilities from vaccine-preventable diseases, the World Health Organization’s Immunisation Agenda 2030 (IA2030) has set a global target of a 50% reduction of zero-dose children by 2030 relative to pre-pandemic levels [1].

Uptake of vaccination depends on many factors beyond access to a health service. The social and economic environment in which the child lives can also affect its vaccination status [1] by limiting opportunities for child vaccination in several ways. For instance, while in upper-middle income countries one may detect the emergence of vaccine hesitancy, in many places poverty and cultural norms can constrain caregivers from seeking health services for their children [3-5]. As women are the primary caregivers in most societies, their empowerment can be critical for achieving better child health outcomes. Women’s empowerment relates to having the autonomy, agency and ability to make informed decisions, including those related to health and regarding when to seek curative or preventive care [6]. The literature has shown that more educated women [7], with greater vaccine knowledge and decision autonomy [6,8] are more likely to have their children vaccinated. Studies specifically addressing the effect of women’s empowerment on child health and vaccination status have been carried out on different populations, usually a single country or a region. The results generally support the hypothesis that women’s empowerment is associated with better child health and higher vaccine coverage [6-15]. There is, however, some inconsistency in the findings from different countries [6-13].

To help provide a global understanding of the role of gender-related barriers to vaccination, we have used a broad measure of women’s empowerment and explored its association with the prevalence of zero-dose children aged 12-23 months across a large number of LMICs, using data from standardized national household surveys. We adopted the Survey-based Women’s Empowerment Global index [16] (SWPER Global) as the measure for women’s empowerment, covering three empowerment domains, and assessed its association with the prevalence of zero-dose within and across countries through pooled analyses.

## METHODS

### Data sources and study sample

Our analyses are based on national health surveys with information on both empowerment scores (using the SWPER Global) and vaccination status [16]. Currently, only Demographic and Health Surveys (DHS) fulfill this requirement. DHS are nationally representative surveys with standardized questionnaires, allowing the comparison of health indicators across countries and over time. For countries with at least one survey conducted since 2010, we selected the most recent one. Our study included children aged 12-23 months - the usual age range for vaccine indicators - and their mothers. For the Dominican Republic (2013), Egypt (2014), and Kyrgyzstan (2012) we studied children aged 18-29 months because measles vaccination takes place after 12 months of age, differently from most countries where it is administered at 9-12 months. Although our outcome does not include measles vaccination, this approach makes our coverage indicator consistent with results from earlier publications [17-19] and from published survey reports. Ethical approval for the conduct of the surveys was obtained by the national institutions involved in data collection. All data used were anonymized.

### Immunisation indicator

For operational purposes, DPT vaccination is used as a proxy of well-functioning routine immunisation programs, with its first dose indicating accessibility to functional health care services [20-22]. For this reason, we used no doses of DPT (or any DPT-containing vaccine) as a proxy to zero-dose children, that is, those failing to receive any routine vaccination as defined by the WHO and the UNICEF [21,23].

We calculated the proportion of children who failed to receive any doses of DPT-containing vaccines, referred to as no-DPT children. The information was collected from the vaccination card and from the mother’s report if information was missing from the card. A child was defined as no-DPT if she/he had no recorded DPT doses on the card and the mother did not report any DPT vaccination or informed that she did not know.

### Women’s empowerment indicator

We measured women’s empowerment using the SWPER Global, an individual-level indicator estimated for women aged 15-49 years who are married or in union [16]. A detailed description of the SWPER indicator and its validity is available elsewhere [16]. It is derived using principal component analyses based on 14 DHS questions covering three domains: a) social independence, related to access to information, education and age of marriage and first birth; b) decision-making, related to making decisions on important household matters; c) attitude towards violence, related to how much the woman rejects domestic violence against the wife. The resulting scores were standardized so that positive values represent above-average levels of empowerment while negative values represent the opposite. The value of zero represents the average empowerment score for all LMICs used to derive the indicator [10,16]. Using the original pooled distribution of the scores, each SWPER Global domain was categorized into low, medium, and high levels of empowerment, based on approximate terciles of the scores.

### Statistical analysis

Ecological analyses with countries as the analytical units relied on Pearson correlation coefficient between no-DPT prevalence and the percentage of women with high level of empowerment. Within-country individual level analyses consisted of calculating the prevalence of no-DPT stratified by empowerment levels (low, medium, and high) for each domain. Finally, pooled individual-level analyses across all countries relied upon multilevel Poisson regression with countries as the second level unit and children as the first level with the intent of evaluating the overall association between maternal empowerment and no-DPT prevalence. Robust Poisson regression was used so that we could estimate prevalence ratios, which are more interpretable than odds ratios resulting from a logistic regression and accounts for overdispersed variance [24]. We also fitted separate sex-specific regression models to assess whether the associations differed among boys and girls. We also carried out sensitivity analyses. The first one aims to verify if the effect of empowerment remains the same after removing countries with low no-DPT prevalence. For this we repeated the ecological analysis excluding countries in the lowest tercile of no-DPT. The second sensitivity analysis aims to remove the effect of potential confounders in the association of empowerment and vaccination: area of residence and wealth. Thus, we estimate the pooled effect adjusting for household wealth quintiles and area of residence (urban/rural). The measurement of wealth was based on an asset index, obtained from information of household appliances, characteristics of the building materials, presence of electricity, water supply and sanitary facilities, among other variables [25,26]. Assets may vary in urban and rural households, separate principal component analyses were carried out in each area, which were later combined into a single score using a scaling procedure to allow comparability between urban and rural households. This score is then divided into quintiles weighted by the number of household members [27]. In the specific case of empowerment and immunisation association, wealth could represent a confounder but also a pathway through which empowerment influences child immunisation. Therefore, the interpretation of adjusted effects should be made with caution. The sensitivity analyses are presented in the supplementary materials.

We estimated two summary measures of inequality, the slope index of inequality (SII) and the concentration index (CIX) [28]. The SII is a logistic regression-based measure of absolute inequality that represents the adjusted difference in the outcome between the children of the most and least empowered women. The SII for prevalence outcomes varies between -100 to 100 percentage points, with negative values representing higher no-DPT prevalence among the children of less empowered women, zero representing perfect equality, and positive values representing higher no-DPT prevalence among the most empowered. The CIX is a measure of relative inequality similar to the Gini index which is frequently used for income inequality. The CIX is calculated as twice the area in a Lorenz curve, varying between -1 and 1. For clarity, we multiply it by 100, negative values meaning the outcome is concentrated among children of less empowered women and positive values meaning the opposite. A CIX of zero means perfect equality in the distribution of the outcome.

We plotted SII vs CIX by terciles of national no-DPT prevalence to evaluate the relationship between the absolute and relative measures of inequality across countries with different vaccination effectiveness.

The analyses were carried out with Stata (StataCorp. 2019. Stata Statistical Software: Release 17. College Station, TX: StataCorp LLC) and R (R Core Team, 2020, version 4.1.0. R Foundation for Statistical Computing, Vienna, Austria) and accounted for the multi-stage survey design. Pooled results were weighted by the national population of children aged 12-23 months in 2015 (the median year of the surveys covered) obtained from the World Bank Population Estimates and Projections [29].

### Ethical aspects

Ethical approval for the conduct of the surveys was obtained by the national institutions involved in data collection. All data used were anonymized.

## RESULTS

We included 50 countries and 94 337 children in the analysis. These countries represent 74% of all low-income, 40% of lower-middle and 11% of the upper-middle-income countries in the world, including 85%, 87% and 8% of all 12-23-month-old children in those regions, respectively. The children included were mostly from rural areas (67.1%) and only 36.2% had mothers in the highest level of the social independence empowerment domain. The median no-DPT prevalence was 9.2% (IQR = 2.7%-17.2%), ranging from 0.5% in Rwanda to 43.3% in Chad. **Table 1** summarizes the sample characteristics.

**Table 1:** Characteristics of the sample, no-DPT prevalence and women’s empowerment levels for 50 countries. Source: DHS 2010-2019

			Unweighted mean (%)	Unweighted SD (%)	Weighted mean (%)	Weighted SD (%)	Range		Median (%)	Interquartile range	
			**Lowest (%)**	**Highest (%)**	**P25 (%)**	**P75 (%)**
Sex of the child		Female	49.1	2.0	48.8	1.6	44.2	55.5	49.2	47.9	50.0
		Male	50.9	2.0	51.2	1.6	44.5	55.8	50.8	50.0	52.1
Place of residence		Rural	64.6	17.1	67.1	11.4	17.8	92.4	67.8	55.7	73.9
		Urban	35.4	17.1	32.9	11.4	7.6	82.2	32.2	26.1	44.3
Immunisation indicator		No-DPT	11.6	11.1	13.0	10.0	0.5	43.3	9.2	2.7	17.2
SWPER domains	Social independence	Low	31.9	19.1	29.1	16.4	0.5	78.2	31.2	14.6	45.6
		Medium	34.7	7.4	34.7	6.0	17.2	50.6	35.1	29.8	39.6
		High	33.4	18.8	36.2	15.5	4.6	77.8	30.9	18.4	44.0
	Decision-making	Low	20.6	18.6	22.1	14.8	1.3	77.7	13.7	6.4	36.7
		Medium	29.7	9.3	27.4	7.5	9.7	50.2	29.9	24.3	34.2
		High	49.7	22.1	50.5	17.5	8.4	87.7	54.8	30.9	66.3
	Attitude towards violence	Low	24.4	17.9	24.2	13.9	0.4	64.0	21.7	11.2	34.9
		Medium	19.2	8.4	19.3	6.7	1.0	40.9	19.7	14.4	25.2
		High	56.4	22.4	56.5	17.3	13.8	98.5	55.2	41.0	68.7

SD – standard deviation, DPT – diphtheria, pertussis, and tetanus, SWPER – Survey-based Women’s Empowerment

**Figure 1:**
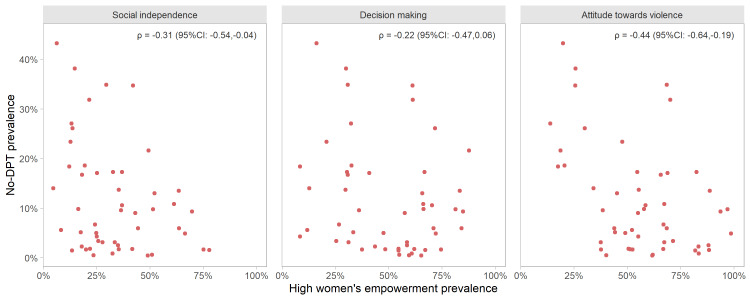
Correlations between no-DPT prevalence and the proportion of women with a high level of empowerment for each SWPER domain, at country level.

In the ecological, country-level analyses we found that the higher the proportion of women in the high level of empowerment, the lower the no-DPT prevalence. **Figure 1** shows weak to moderate negative correlations between the two variables, with correlation coefficients of -0.44 (95% confidence interval (CI) = -0.64, -0.19) for the attitude towards violence domain, -0.31 (95% CI = -0.54, -0.04) for the social independence domain and -0.22 (95% CI = -0.47, 0.06) for the decision-making domain. The analysis excluding countries in the lowest tercile of no-DPT had very similar correlation coefficients for attitude towards violence, decision-making and social independence, namely -0.41, -0.22 and -0.38 (Figure S4 in the **Online Supplementary Document**).

**Figure 2:**
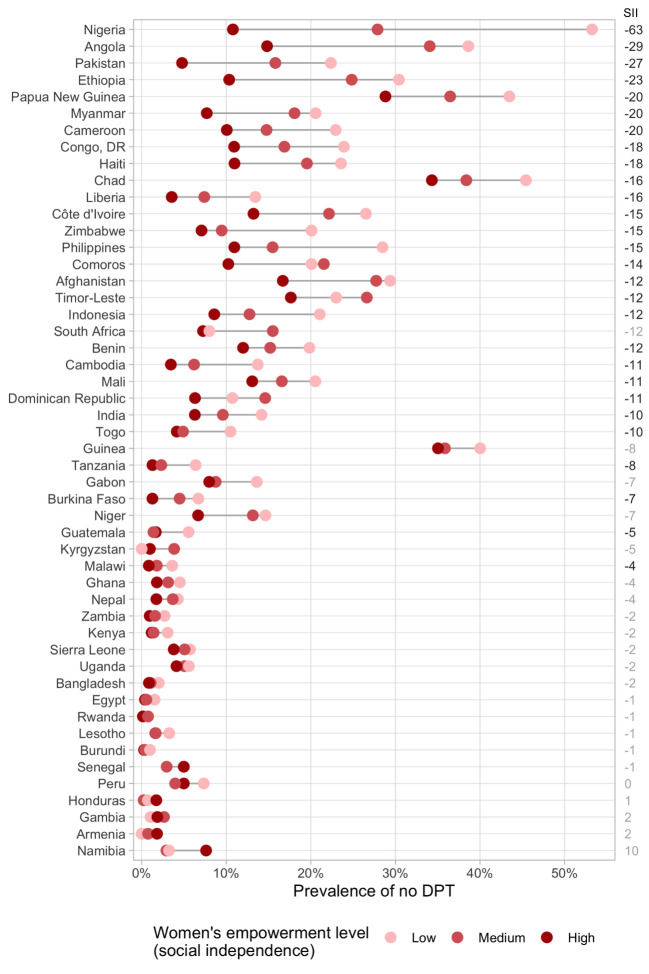
No-DPT prevalence according to levels of SWPER’s social independence domain by country. Light grey font indicates non-significant SII values. Note: countries are ordered by the slope index of inequality (SII). Estimates with N<25: Armenia (low), Kyrgyzstan(low) and South Africa (low).

In the individual level analyses, overall, children of highly-empowered mothers presented lower prevalence of no-DPT than those of less-empowered mothers. The social independence domain presented more consistent associations with no-DPT compared to the other two domains. In 42 out of the 50 countries, the lowest point estimate for no-DPT prevalence was in the high empowerment groups, with the slope index of inequality showing significant results in 28 countries. Also, the eight countries where this was not the case were among the 20 with the lowest levels of no-DPT, where differences across groups were rather small. Prevalence estimates of no-DPT according to the level of empowerment in the social independence domain are presented in **Figure 2** for each of the study countries. Results for the attitude towards violence and decision-making domains are presented in the supplemental material, Figures S1 and S2 the **Online Supplementary Document**, respectively.

For social independence, large inequalities in no-DPT prevalence were observed within many countries, especially those with high overall prevalence. Nigeria, Angola, Pakistan, and Ethiopia presented the highest levels of inequality in this domain with SIIs of -63, -29, -27 and -23 percentage points (pp), respectively. Nigeria was also the most inequitable country both in terms of attitude towards violence (SII = -45 pp, Figure S1 in the **Online Supplementary Document**) and decision-making (SII = -47 pp, in the **Online Supplementary Document**) domains.

**Figure 3:**
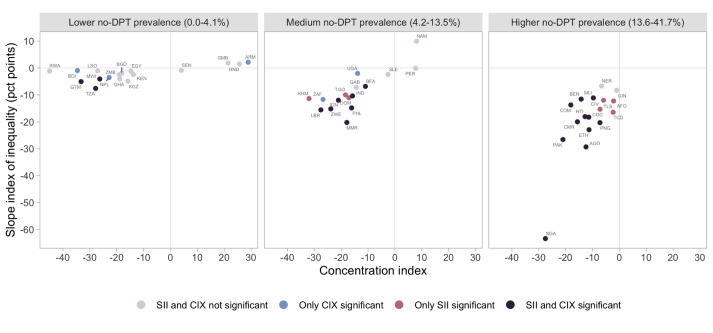
Absolute and relative inequalities relative to women’s empowerment, using the social independence domain of the SWPER. Countries identified by the 3-letter ISO code (see Table S1 in the **Online Supplementary Document**). SII and CIX significant when *P* < 0.05. Countries were dived into three panels, according to terciles of no-DPT prevalence among all children in the country.

We compared countries in terms of both absolute inequality (SII) and relative inequality (CIX) after grouping countries by terciles of no-DPT prevalence. The graph on the right side of **Figure 3** shows that countries in the highest tercile presented the highest absolute inequalities, but also high relative inequalities, with consistent inverse associations as shown by the negative values for SII and CIX. A similar situation was observed in the intermediate tercile of no-DPT (graph in the middle of **Figure 3**). On the left side of this figure, countries with the lowest no-DPT prevalence showed very small absolute inequalities – as expected – along with high relative inequalities which most often were in favour of more empowered women. In four countries (out of 17) the CIX was positive, with no-DPT more concentrated towards more empowered women, but in none of these countries the CIX was statistically significant.

**Figure 4:**
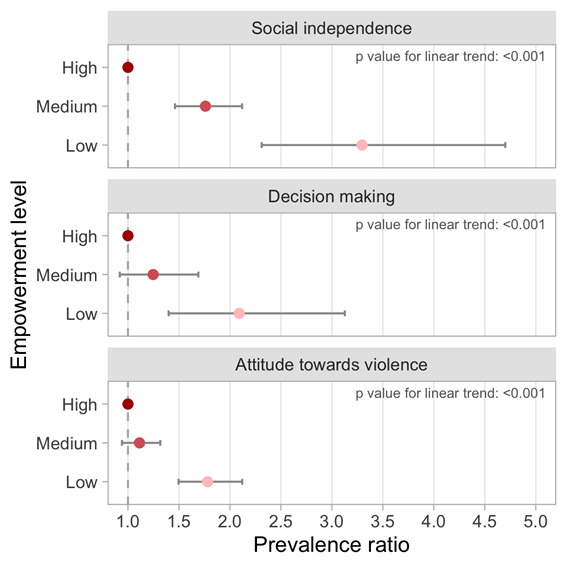
Pooled prevalence ratio for no-DPT according to level of empowerment for each SWPER domain.

When we pooled all countries using a multilevel Poisson model, children from mothers in the low and medium levels of the social independence domain had 3.3 (95% CI = 2.3, 4.7) and 1.8 (95% CI = 1.5, 2.1) times higher no-DPT prevalence compared to those in the high level (**Figure 4**). Table S3 in the **Online Supplementary Document** shows the prevalence ratio adjusted for wealth quintiles and area of residence. Although the magnitude is reduced after adjustment, it remains high and statistically significant (low = 2.5 (95% CI = 1.8, 3.4); medium = 1.4 (95% CI = 1.2, 1.7). For the decision-making and attitude to violence domains, prevalence of no-DPT was higher in the lowest empowerment level when compared to the high level: 2.1 times (95% CI = 1.4, 3.1) and 1.8 times higher (95% CI = 1.5, 2.1), respectively. For these two domains, there were no strong differences between the intermediate and lowest empowerment levels, as the 95% confidence intervals included the unity: 1.2 times (95% CI = 0.9, 1.7) for decision-making and 1.1 times (95% CI = 0.9, 1.3) for attitude towards violence.

We found no difference in the effect of empowerment on no-DPT prevalence by child’s sex. The interaction term between empowerment and sex presented a *P* = 0.192 for social independence domain, *P* = 0.637 for the decision-making domain and *P* = 0.157 for the attitude towards violence domain (Figure S3 in the **Online Supplementary Document**).

## DISCUSSION

Our analyses included a wide range of LMICs representing 74% and 40% of all low- and lower-middle-income countries, respectively. We used a zero-dose indicator based on children who failed to receive any doses of DPT to explore how women’s empowerment might affect routine immunisation. Our results showed that children born to less empowered women are over three times more likely to belong to the zero-dose category compared to those born to women with a high level of empowerment in the social independence domain. The other two domains showed important, albeit smaller associations. The absolute gaps were more marked in countries with high zero-dose prevalence such as Nigeria, Angola, Ethiopia, and Pakistan.

The fact that inequalities, both absolute and relative, are particularly large in countries with high levels of zero-dose suggests that less effective immunisation programs contribute to increasing inequalities given they require stronger engagement by mothers in order to get their children vaccinated. While we found associations with all three domains under study, these were stronger for the social independence domain. This suggests that aspects of empowerment related to autonomy and agency may be more relevant for achieving child immunisation than maternal traits related to decision making or attitude to violence.

Our findings are consistent with the existing literature, which has reported positive associations between women’s empowerment and improved child health [6,13]. In addition, two systematic reviews addressed this topic. Pratley (2016) combined evidence from LMICs and concluded that although individual studies may differ in magnitude and, in some cases, direction, there is evidence to support that women’s empowerment is positively associated with health outcomes [13]. More recently, Abreha et al. reviewed the evidence from sub-Saharan Africa and also concluded that women’s empowerment, especially decision making and autonomy, was positively associated with child health outcomes [6]. Our study contributes to the literature by using consistent definitions of empowerment and zero-dose, and standardized analytical approaches in 50 LMICs.

According to the highly-cited work by Kabeer [30], empowerment is the process that enables women who have been denied the ability to make strategic life choices to acquire such an ability. Given its multidimensional nature, attempts to measure empowerment consider the degree of access to and control over material and social resources within the family, community, and society. Although many factors besides maternal empowerment might affect the access and use of health services, particularly vaccination, we found that increased empowerment in three domains was associated with a lower frequency of non-vaccination. This suggests that promoting maternal empowerment would enable to effectively access existing services for themselves and for their children, and therefore positively impact their health and welfare [31]. Assuming that this association is causal, our results show that there would be 4.7 million fewer no-DPT children in the world if all of them had empowered mothers.

In our analyses, the more evident association was found for the social independence domain. Looking at the variables used for the construction of the social independence domain (as discussed in the Methods section) it is reasonable to expect that women with more access to information and who did not have to interrupt their education or careers because of marriage/birth in adolescence might have children with better health outcomes.

On other hand, the questions used for the construction of the decision-making domain are related to the respondent’s own health care, large household purchases and visits to family or relatives. Therefore, it is reasonable to expect a weaker association with child health. This is confirmed by the results of other research [32,33].

It is not surprising that no differences were observed in the effects of empowerment on zero-dose prevalence among boys and girls. In most countries, sex inequalities in early life tend to be small and – when present – to favour girls given the greater biological frailty of boys [34,35]. There are clear context specific exceptions – such as higher than expected male-to-female sex ratios in India due to preferences for sons – but prevalence of zero-dose has been found to be quite similar between boys and girls in LMICs [18,34].

This work comprises a multi-country analysis with standardized methodology and questionnaires, which allowed a broad view of the issue through pooled results and country-specific estimates. It also makes possible comparison of countries with different contexts and cultures. Except for the two literature reviews mentioned above, we were unable to located any multi-country studies on women’s empowerment and child immunisation in different regions of the world, as the existing literature is usually limited to particular sets of countries [9,11,12,31]. Specifically for vaccination coverage, existing studies are usually restricted to population of single countries [14,15,36]. The published study with the biggest set of countries (26 countries from sub-Saharan Africa) also found results in the same direction from our analyses [12]. Our broad multi-country analyses were made possible by the use of a validated and standardized measure of women’s empowerment, the SWPER Global [16].

Some limitations need to be mentioned. Vaccination status is based on retrospective information for children, relying on the availability of a vaccine card and on maternal recall. Women’s empowerment may be associated with enhanced recall, or a greater likelihood of having a vaccine card available, thus possibly inflating the observed gaps and associations. Also, restricting the analyses to children aged 12-23 months as internationally recommended means that many children received their first dose in the past, while empowerment is defined mostly by present-time indicators. Thus, the current level of empowerment may have been different relative to when vaccination was due. Although we have chosen a zero-dose definition used by the UNICEF and the WHO (those who received no doses of DTP [21,23]) and other studies [37,38], it is not a complete zero-dose. The idea behind the operationalization of zero-dose taking into account only DPT is because having no doses of DPT is a good proxy of poor access to routine immunization and other health services. In addition, DPT is considered a basic vaccine in most countries. The definition of “zero-dose” varies. A complete zero-dose that would take into account all the basic vaccines in a country would, by definition, vary according to the national immunization calendar. For example, a complete zero-dose in Ethiopia would include 10 different vaccines. Therefore, no-DPT tends to improve the comparison between countries and is proposed by Global Vaccine Action Plan as a target for monitoring immunization [39]. An important limitation of the SWPER is its restriction to currently married women, given that some key questions are not applied to unpartnered women whose children are thus excluded from our analyses. Finally, differences between countries for both empowerment and vaccination could be better understood taking into account context-specific characteristics of each country such as health service structure and cultural norms, which are not addressed in our analyses. Our results warrant further research in order to investigate such context specific characteristics that may explain our findings and further subsidise local policy makers.

In summary, our country-level and individual-level analyses supports the importance of women’s empowerment for child vaccination, especially where health systems needed for routine vaccination are weaker. Making progress towards the ambitious zero-dose target of Immunisation Agenda 2030 will require focused efforts to address and overcome gender-related barriers to immunisation.

## Additional material


Online Supplementary Document
