# Supplementary material for: Children of more empowered women are less likely to be left without vaccination in low- and middle-income countries: A global analysis of 50 DHS surveys
Source: J Glob Health. 2022 Mar 26;12:04022. doi: 10.7189/jogh.12.04022 (PMC8943525; doi:10.7189/jogh.12.04022)
Supplement: Online Supplementary Document [file jogh-12-04022-s001.pdf]

Supplementary material

Table S1 – Countries and estimates

					No-DPT prevalence (CI 95%)									
Country	ISO	No-DPT prevalence (CI 95%)		SWPER domain	Low		Medium		High		SII	P value	CIX	P value
				Social independence	29.4	(25.4,33.7)	27.7	(23.3,32.7)	16.7	(11.9,22.9)	-12.2	0.012	-2.1	0.496
Afghanistan	AFG	27.1	(23.8,30.7)	Decision making	36.2	(30.3,42.6)	22.5	(18.1,27.7)	21.0	(18.2,24.1)	-23.2	0.000	-10.3	0.000
				Attitude towards violence	28.7	(24.5,33.2)	26.8	(22.4,31.7)	21.7	(18.0,25.9)	-7.9	0.038	-0.6	0.761
				Social independence	38.6	(33.9,43.6)	34.1	(28.9,39.7)	14.8	(10.8,20.0)	-29.3	0.000	-12.4	0.000
Angola	AGO	31.8	(28.6,35.2)	Decision making	46.0	(35.0,57.4)	32.2	(27.3,37.5)	30.1	(26.1,34.5)	-11.0	0.066	-2.8	0.368
				Attitude towards violence	40.9	(32.5,49.9)	39.8	(32.7,47.4)	28.2	(24.8,31.9)	-21.9	0.000	-6.0	0.046
				Social independence	0.0		0.8	(0.1,5.3)	1.8	(0.8,4.1)	2.2	0.332	28.9	0.046
Armenia	ARM	1.6	(0.7,3.4)	Decision making	0.0		3.7	(1.4,9.7)	0.7	(0.2,2.3)	-4.1	0.134	-14.5	0.559
				Attitude towards violence	3.2	(0.4,20.7)	0.0		1.6	(0.7,3.7)	-0.1	0.975	21.9	0.273
				Social independence	2.0	(1.2,3.5)	1.1	(0.4,2.5)	0.9	(0.3,2.7)	-1.9	0.085	-18.2	0.114
Bangladesh	BGD	1.5	(0.9,2.5)	Decision making	1.6	(0.5,5.6)	2.3	(1.2,4.4)	0.9	(0.5,1.8)	-1.8	0.209	-18.0	0.146
				Attitude towards violence	0.0		2.4	(0.9,6.0)	1.4	(0.8,2.2)	-0.9	0.515	-8.4	0.427
				Social independence	19.8	(16.7,23.5)	15.2	(12.5,18.3)	12.0	(8.9,15.9)	-11.5	0.002	-14.2	0.000
Benin	BEN	16.7	(14.7,19.0)	Decision making	24.0	(20.1,28.4)	16.4	(13.6,19.6)	9.9	(7.5,12.9)	-20.1	0.000	-22.5	0.000
				Attitude towards violence	20.5	(16.4,25.3)	15.7	(11.6,20.8)	15.8	(13.5,18.3)	-6.2	0.086	-9.3	0.006
	BFA	5.6	(4.5,7.1)	Social independence	6.7	(5.2,8.6)	4.5	(2.7,7.3)	1.3	(0.4,3.9)	-6.8	0.008	-11.0	0.032

Burkina Faso				Decision making	3.5	(2.4,5.2)	7.9	(5.9,10.3)	4.9	(2.7,8.9)	5.3	0.009	14.9	0.005
				Attitude towards violence	5.4	(3.6,8.2)	5.0	(3.0,8.1)	6.0	(4.5,8.0)	1.2	0.559	3.8	0.544
				Social independence	1.0	(0.4,2.4)	0.4	(0.1,1.1)	0.3	(0.1,1.1)	-0.9	0.154	-34.6	0.016
Burundi	BDI	0.5	(0.3,0.9)	Decision making	0.0		0.7	(0.3,1.8)	0.6	(0.3,1.2)	0.4	0.471	-3.8	0.781
				Attitude towards violence	0.5	(0.2,1.5)	0.3	(0.1,1.1)	0.7	(0.3,1.6)	0.3	0.609	0.8	0.960
				Social independence	13.7	(8.5,21.3)	6.2	(4.0,9.5)	3.5	(1.9,6.3)	-11.3	0.003	-31.9	0.000
Cambodia	KHM	6.0	(4.5,7.9)	Decision making	0.8	(0.1,6.2)	2.7	(1.2,6.4)	6.6	(4.9,8.9)	8.5	0.032	-1.4	0.864
				Attitude towards violence	8.2	(5.1,13.1)	5.3	(3.2,8.8)	5.2	(3.2,8.3)	-3.9	0.189	-15.3	0.053
				Social independence	22.9	(18.3,28.4)	14.8	(11.3,19.0)	10.1	(6.7,14.9)	-20.0	0.000	-15.6	0.000
Cameroon	CMR	17.1	(14.3,20.3)	Decision making	24.0	(18.7,30.4)	12.2	(8.4,17.4)	13.6	(10.6,17.2)	-16.4	0.003	-13.0	0.005
				Attitude towards violence	23.1	(16.3,31.6)	15.3	(10.6,21.5)	16.1	(13.1,19.7)	-7.2	0.148	-2.1	0.624
				Social independence	45.4	(42.0,48.9)	38.4	(33.5,43.5)	34.3	(23.8,46.6)	-16.4	0.004	-2.3	0.225
Chad	TCD	43.3	(40.1,46.4)	Decision making	48.8	(44.2,53.5)	40.1	(35.9,44.5)	38.1	(32.5,44.0)	-16.5	0.001	-4.3	0.032
				Attitude towards violence	41.6	(37.8,45.4)	42.3	(36.5,48.4)	49.3	(43.2,55.4)	10.0	0.067	4.7	0.028
				Social independence	20.1	(14.1,27.7)	21.5	(14.5,30.8)	10.2	(6.3,16.1)	-13.7	0.028	-18.1	0.004
Comoros	COM	17.3	(13.5,22.0)	Decision making	22.4	(16.0,30.4)	12.6	(6.9,21.8)	16.3	(10.3,25.0)	-10.1	0.191	-12.9	0.067
				Attitude towards violence	25.1	(16.9,35.6)	19.4	(11.7,30.4)	13.4	(9.2,19.3)	-17.0	0.038	-17.2	0.035
				Social independence	23.9	(20.5,27.6)	16.9	(13.4,21.0)	10.9	(7.4,15.9)	-18.3	0.000	-11.4	0.002
Congo, DR	COD	18.6	(16.3,21.2)	Decision making	23.6	(19.3,28.4)	18.0	(14.7,21.8)	15.0	(12.3,18.3)	-11.8	0.001	-7.2	0.022
				Attitude towards violence	21.2	(17.7,25.3)	16.5	(12.9,21.0)	15.0	(11.3,19.6)	-10.0	0.026	-4.4	0.158
				Social independence	26.5	(21.1,32.7)	22.1	(16.0,29.8)	13.2	(7.8,21.6)	-15.3	0.027	-7.2	0.116
Côte D'Ivoire	CIV	23.4	(19.5,27.8)	Decision making	26.2	(20.5,32.8)	23.6	(17.4,31.4)	16.7	(11.7,23.1)	-12.4	0.047	-6.3	0.149
				Attitude towards violence	27.9	(21.0,36.1)	27.7	(20.2,36.6)	18.5	(13.9,24.2)	-15.9	0.021	-7.6	0.130

Dominican Republic	DOM	9.8	(6.6,14.3)	Social independence	10.7	(5.6,19.7)	14.6	(9.0,22.8)	6.3	(3.1,12.3)	-11.0	0.033	-17.1	0.067
				Decision making	0.0		10.3	(4.5,22.1)	10.0	(6.4,15.4)	2.9	0.746	4.5	0.694
				Attitude towards violence	29.5	(3.3,83.6)	35.9	(9.4,75.2)	9.0	(5.8,13.8)	-34.5	0.088	-5.2	0.706
Egypt	EGY	0.6	(0.3,1.1)	Social independence	1.5	(0.6,3.8)	0.6	(0.2,1.5)	0.4	(0.1,1.0)	-1.2	0.160	-14.7	0.408
				Decision making	1.4	(0.3,5.4)	0.2	(0.1,0.8)	0.7	(0.3,1.4)	0.0	0.966	15.0	0.472
				Attitude towards violence	1.2	(0.4,3.6)	0.6	(0.2,2.0)	0.4	(0.2,1.0)	-1.0	0.245	-8.5	0.680
Ethiopia	ETH	26.1	(22.5,30.1)	Social independence	30.4	(25.8,35.5)	24.8	(18.8,32.0)	10.3	(5.7,18.1)	-22.9	0.000	-11.3	0.003
				Decision making	33.6	(24.1,44.6)	22.2	(16.5,29.2)	26.0	(21.5,31.0)	-2.8	0.699	-0.9	0.819
				Attitude towards violence	27.8	(22.4,33.9)	22.9	(16.3,31.3)	25.9	(20.5,32.2)	-4.0	0.560	-1.6	0.713
Gabon	GAB	9.6	(6.7,13.5)	Social independence	13.6	(8.0,22.3)	8.7	(5.3,14.1)	8.0	(3.8,16.1)	-7.2	0.243	-14.4	0.185
				Decision making	11.1	(4.6,24.4)	11.0	(6.9,17.0)	7.1	(4.1,11.9)	-6.4	0.274	-13.1	0.112
				Attitude towards violence	15.5	(8.6,26.2)	4.5	(2.6,7.7)	10.4	(6.2,17.0)	-4.7	0.503	-10.7	0.287
Gambia	GMB	1.7	(1.0,2.9)	Social independence	1.0	(0.4,2.5)	2.6	(1.2,5.5)	1.9	(0.7,5.0)	1.9	0.265	21.4	0.141
				Decision making	3.9	(1.4,10.6)	1.5	(0.7,3.2)	0.8	(0.4,1.9)	-3.8	0.124	-22.8	0.154
				Attitude towards violence	1.4	(0.7,2.6)	2.6	(1.1,6.1)	1.3	(0.3,5.0)	-0.2	0.896	4.2	0.769
Ghana	GHA	3.1	(2.0,4.9)	Social independence	4.5	(2.5,8.0)	3.1	(1.4,7.0)	1.8	(0.8,3.9)	-4.0	0.068	-18.9	0.058
				Decision making	5.2	(2.1,11.9)	6.2	(3.4,11.2)	1.1	(0.5,2.3)	-8.6	0.001	-35.4	0.000
				Attitude towards violence	3.4	(0.8,13.3)	6.8	(3.2,13.8)	2.1	(1.2,3.8)	-4.7	0.136	-16.3	0.230
Guatemala	GTM	2.6	(1.9,3.5)	Social independence	5.5	(3.7,8.2)	1.4	(0.8,2.4)	1.7	(0.7,3.8)	-5.0	0.012	-33.3	0.001
				Decision making	3.9	(1.7,8.6)	3.3	(1.9,5.6)	2.0	(1.3,3.1)	-2.5	0.111	-14.9	0.132
				Attitude towards violence	1.0	(0.1,6.8)	3.5	(1.7,7.1)	2.5	(1.8,3.6)	-0.3	0.860	-1.6	0.846
Guinea	GIN	38.1	(34.3,42.1)	Social independence	40.0	(35.2,45.0)	35.9	(29.9,42.3)	35.0	(27.7,43.1)	-8.3	0.144	-1.0	0.685
				Decision making	45.3	(40.2,50.5)	37.3	(30.9,44.2)	29.9	(24.7,35.6)	-22.8	0.000	-7.1	0.004

				Attitude towards violence	42.1	(37.2,47.3)	30.4	(23.0,39.1)	33.8	(27.5,40.8)	-16.5	0.015	-3.3	0.216
				Social independence	23.6	(17.4,31.1)	19.5	(14.6,25.6)	11.0	(7.1,16.6)	-18.1	0.002	-12.8	0.014
Haiti	HTI	17.3	(13.9,21.3)	Decision making	20.2	(11.6,32.9)	16.9	(12.0,23.4)	17.1	(13.2,21.9)	-1.7	0.778	2.3	0.708
				Attitude towards violence	20.2	(11.0,34.2)	10.6	(5.8,18.5)	18.1	(14.4,22.5)	8.8	0.236	10.6	0.133
				Social independence	0.7	(0.3,2.0)	0.2	(0.0,1.6)	1.7	(0.8,3.8)	1.5	0.197	25.7	0.140
Honduras	HND	0.9	(0.5,1.6)	Decision making	2.5	(0.5,10.7)	0.0		1.2	(0.6,2.4)	1.6	0.164	23.3	0.117
				Attitude towards violence	0.0		0.9	(0.2,4.1)	0.9	(0.5,1.8)	0.6	0.619	6.5	0.645
				Social independence	14.2	(12.2,16.4)	9.6	(8.4,11.0)	6.3	(5.1,7.7)	-10.4	0.000	-15.8	0.000
India	IND	9.1	(8.2,10.0)	Decision making	12.6	(10.4,15.2)	8.7	(7.2,10.5)	8.0	(7.0,9.1)	-6.0	0.001	-7.4	0.013
				Attitude towards violence	11.6	(9.8,13.6)	8.1	(6.6,9.8)	8.3	(7.2,9.6)	-4.2	0.012	-5.6	0.046
				Social independence	21.1	(15.0,28.7)	12.7	(10.2,15.8)	8.6	(7.2,10.1)	-12.0	0.000	-21.0	0.000
Indonesia	IDN	10.8	(9.5,12.4)	Decision making	16.2	(9.8,25.6)	12.1	(9.6,15.0)	9.9	(8.5,11.6)	-5.2	0.039	-14.7	0.000
				Attitude towards violence	14.2	(9.7,20.2)	10.2	(8.0,12.8)	10.7	(9.2,12.5)	-1.3	0.596	-10.2	0.003
				Social independence	3.1	(1.8,5.2)	1.4	(0.8,2.6)	1.2	(0.5,2.7)	-2.4	0.057	-13.8	0.230
Kenya	KEN	1.8	(1.2,2.6)	Decision making	2.2	(0.9,5.1)	1.3	(0.7,2.4)	2.0	(1.1,3.5)	0.5	0.707	14.9	0.227
				Attitude towards violence	2.8	(1.5,5.3)	1.3	(0.6,2.8)	1.5	(0.9,2.7)	-1.3	0.285	-3.2	0.789
				Social independence	0.0		3.8	(1.4,10.4)	1.0	(0.3,3.2)	-4.9	0.154	-15.8	0.545
Kyrgyzstan	KGZ	1.7	(0.8,3.6)	Decision making	0.0		4.0	(1.1,13.7)	1.2	(0.5,3.2)	-2.7	0.405	-9.4	0.676
				Attitude towards violence	1.4	(0.5,3.8)	1.6	(0.5,5.2)	1.8	(0.5,5.9)	0.6	0.785	17.7	0.397
				Social independence	3.3	(0.5,19.0)	1.7	(0.6,4.4)	1.6	(0.6,4.7)	-1.1	0.694	-27.1	0.115
Lesotho	LSO	1.8	(0.9,3.5)	Decision making	2.6	(0.4,17.3)	1.9	(0.6,5.6)	1.7	(0.7,4.2)	-0.7	0.778	-26.6	0.009
				Attitude towards violence	0.0		0.4	(0.1,3.1)	2.5	(1.3,5.0)	5.6	0.059	3.7	0.775
Liberia	LBR	9.9	(7.8,12.5)	Social independence	13.4	(9.8,18.1)	7.4	(4.7,11.4)	3.6	(1.8,6.9)	-15.6	0.002	-27.6	0.000

				Decision making	10.2	(4.4,21.8)	10.1	(6.2,16.0)	9.7	(7.0,13.4)	-0.7	0.896	-7.7	0.289
				Attitude towards violence	11.4	(7.0,17.8)	16.2	(11.0,23.2)	7.4	(5.0,10.9)	-8.7	0.066	-16.3	0.038
				Social independence	3.6	(2.3,5.6)	1.8	(1.2,2.7)	0.8	(0.2,3.1)	-4.1	0.011	-26.3	0.002
Malawi	MWI	2.3	(1.7,3.1)	Decision making	2.8	(1.4,5.4)	2.6	(1.5,4.3)	1.9	(1.2,3.0)	-1.3	0.310	-8.6	0.369
				Attitude towards violence	2.0	(0.7,5.9)	4.5	(1.9,10.4)	2.0	(1.5,2.8)	-2.8	0.203	-17.6	0.063
				Social independence	20.5	(16.7,25.0)	16.6	(13.0,20.9)	13.1	(8.6,19.3)	-11.1	0.018	-9.7	0.014
Mali	MLI	18.4	(15.5,21.8)	Decision making	19.2	(16.0,22.8)	18.6	(13.7,24.8)	11.7	(6.8,19.3)	-5.9	0.272	-5.6	0.170
				Attitude towards violence	18.3	(14.5,22.8)	17.2	(13.2,22.1)	20.4	(15.3,26.7)	1.6	0.744	-1.8	0.697
				Social independence	20.6	(12.9,31.2)	18.1	(12.4,25.5)	7.7	(4.9,11.8)	-20.2	0.000	-17.9	0.004
Myanmar	MMR	13.0	(9.9,16.9)	Decision making	16.4	(8.2,30.0)	13.0	(8.1,20.2)	12.7	(9.0,17.7)	-2.4	0.705	5.7	0.492
				Attitude towards violence	14.2	(8.0,24.1)	13.0	(8.9,18.6)	12.7	(8.7,18.0)	-1.6	0.771	2.3	0.714
				Social independence	3.2	(0.8,11.5)	2.9	(0.5,14.2)	7.6	(4.3,13.1)	10.0	0.166	8.0	0.445
Namibia	NAM	5.9	(3.6,9.6)	Decision making	6.5	(0.9,34.5)	7.2	(2.9,16.6)	5.5	(2.9,10.3)	-2.6	0.692	-15.3	0.246
				Attitude towards violence	1.9	(0.6,6.4)	7.1	(2.0,22.1)	6.5	(3.6,11.3)	4.8	0.462	6.7	0.632
				Social independence	4.3	(2.5,7.4)	3.7	(1.9,7.0)	1.7	(0.6,5.3)	-3.5	0.160	-22.8	0.031
Nepal	NPL	3.4	(2.3,5.0)	Decision making	2.3	(1.1,4.5)	4.0	(2.0,8.0)	4.5	(2.3,8.9)	3.6	0.155	7.9	0.507
				Attitude towards violence	4.9	(1.6,14.1)	5.2	(2.3,11.3)	2.7	(1.6,4.5)	-4.1	0.164	-25.0	0.020
				Social independence	14.6	(12.2,17.4)	13.1	(9.4,18.0)	6.7	(2.1,19.3)	-6.7	0.122	-6.6	0.142
Niger	NER	14.0	(11.7,16.7)	Decision making	16.5	(13.5,19.9)	11.3	(8.2,15.2)	10.6	(6.6,16.5)	-10.4	0.006	-11.7	0.004
				Attitude towards violence	14.2	(10.9,18.2)	12.1	(8.4,17.2)	14.6	(11.4,18.6)	0.4	0.927	-1.0	0.854
				Social independence	53.2	(50.4,56.1)	27.9	(25.0,31.0)	10.8	(9.1,12.8)	-63.4	0.000	-27.5	0.000
Nigeria	NGA	34.9	(32.9,36.9)	Decision making	49.0	(46.1,52.0)	34.3	(31.2,37.6)	17.0	(14.7,19.6)	-46.6	0.000	-22.2	0.000
				Attitude towards violence	53.7	(49.4,58.0)	46.0	(40.5,51.5)	27.1	(25.1,29.3)	-45.3	0.000	-21.8	0.000

Pakistan	PAK	13.7	(11.3,16.6)	Social independence	22.4	(17.6,28.1)	15.8	(11.5,21.4)	4.8	(3.1,7.2)	-26.6	0.000	-21.0	0.000
				Decision making	17.6	(14.1,21.8)	13.0	(8.9,18.7)	8.3	(5.5,12.5)	-16.1	0.001	-6.6	0.143
				Attitude towards violence	22.7	(17.7,28.7)	12.8	(8.2,19.5)	9.0	(6.7,12.0)	-20.7	0.000	-13.5	0.013
Papua New Guinea	PNG	34.7	(30.9,38.8)	Social independence	43.5	(36.0,51.3)	36.5	(30.6,42.8)	28.8	(23.3,35.0)	-20.3	0.003	-7.2	0.022
				Decision making	46.3	(34.1,58.9)	29.9	(23.9,36.8)	35.1	(30.5,40.0)	-2.2	0.763	1.2	0.683
				Attitude towards violence	39.6	(34.3,45.3)	27.9	(21.6,35.2)	32.2	(26.1,39.1)	-14.6	0.023	-4.4	0.121
Peru	PER	4.9	(3.8,6.3)	Social independence	7.3	(4.6,11.5)	4.0	(2.7,5.7)	5.0	(3.5,7.1)	-0.1	0.977	7.8	0.336
				Decision making	10.8	(5.9,18.8)	7.3	(4.2,12.6)	3.8	(2.9,4.9)	-7.1	0.024	-12.5	0.060
				Attitude towards violence	4.3	(0.9,18.4)	12.6	(3.9,33.7)	4.8	(3.7,6.3)	-7.7	0.271	14.5	0.032
Philippines	PHL	13.5	(11.6,15.7)	Social independence	28.5	(20.5,38.1)	15.5	(11.8,20.0)	11.0	(8.7,13.7)	-14.8	0.000	-16.1	0.001
				Decision making	19.6	(10.9,32.7)	8.5	(5.3,13.2)	14.2	(12.0,16.8)	8.9	0.105	1.3	0.797
				Attitude towards violence	16.4	(7.0,33.8)	15.3	(9.4,23.9)	13.3	(11.2,15.7)	-4.1	0.523	-5.0	0.355
Rwanda	RWA	0.5	(0.2,1.2)	Social independence	0.8	(0.1,5.3)	0.7	(0.2,2.6)	0.1	(0.0,1.0)	-1.1	0.165	-45.0	0.064
				Decision making	1.6	(0.2,10.4)	0.7	(0.2,2.3)	0.2	(0.1,0.9)	-1.2	0.119	-44.0	0.070
				Attitude towards violence	0.5	(0.1,3.4)	1.1	(0.4,3.3)	0.2	(0.1,0.9)	-0.9	0.298	-41.2	0.008
Senegal	SEN	4.3	(2.9,6.4)	Social independence	5.0	(2.9,8.7)	3.0	(1.6,5.4)	5.0	(2.1,11.2)	-0.9	0.814	4.0	0.715
				Decision making	5.0	(3.3,7.7)	2.5	(0.8,7.4)	0.5	(0.1,3.6)	-12.3	0.106	-5.1	0.648
				Attitude towards violence	5.3	(2.9,9.4)	1.1	(0.2,5.0)	4.2	(2.3,7.3)	-1.3	0.694	0.8	0.941
Sierra Leone	SLE	5.2	(3.9,6.8)	Social independence	5.7	(4.0,8.2)	5.1	(3.2,7.9)	3.8	(2.1,6.8)	-2.4	0.274	-2.6	0.686
				Decision making	3.7	(2.4,5.8)	7.0	(4.6,10.7)	5.8	(3.4,9.5)	3.6	0.186	14.2	0.071
				Attitude towards violence	4.7	(3.2,6.9)	3.9	(2.0,7.5)	6.0	(3.9,9.1)	2.2	0.413	9.9	0.276
South Africa	ZAF	9.3	(5.5,15.4)	Social independence	8.0	(1.1,41.0)	15.5	(6.3,33.4)	7.3	(3.5,14.5)	-11.7	0.266	-26.7	0.020
				Decision making	31.2	(8.2,69.7)	2.8	(0.6,13.0)	10.1	(5.8,17.0)	10.7	0.364	-4.5	0.757

				Attitude towards violence	13.9	(1.8,59.2)	6.4	(0.8,35.1)	9.4	(5.4,15.9)	2.9	0.835	-11.5	0.469
				Social independence	6.4	(4.3,9.3)	2.3	(1.2,4.3)	1.3	(0.6,2.8)	-7.6	0.000	-27.9	0.000
Tanzania	TZA	3.2	(2.3,4.4)	Decision making	8.4	(5.3,13.0)	2.0	(1.1,3.4)	2.0	(1.0,3.8)	-7.6	0.006	-25.1	0.015
				Attitude towards violence	4.4	(2.8,6.8)	1.8	(0.9,3.6)	2.6	(1.6,4.1)	-3.0	0.122	-7.1	0.419
				Social independence	23.0	(17.1,30.2)	26.6	(21.9,31.9)	17.6	(14.6,21.1)	-12.0	0.006	-5.9	0.074
Timor-Leste	TLS	21.6	(18.8,24.7)	Decision making	42.6	(29.3,57.2)	29.2	(20.7,39.4)	20.1	(17.3,23.4)	-20.7	0.005	0.1	0.988
				Attitude towards violence	22.8	(19.3,26.8)	15.8	(11.3,21.6)	22.9	(17.0,30.1)	-4.5	0.445	0.6	0.872
				Social independence	10.5	(7.4,14.7)	4.9	(3.1,7.6)	4.1	(1.7,9.7)	-10.0	0.006	-18.4	0.056
Togo	TGO	6.7	(4.8,9.4)	Decision making	9.1	(5.7,14.2)	6.0	(4.0,8.9)	5.3	(2.8,9.9)	-5.3	0.129	-8.7	0.358
				Attitude towards violence	5.9	(2.5,13.2)	7.0	(3.8,12.4)	6.9	(5.0,9.5)	1.2	0.737	4.5	0.522
				Social independence	5.6	(3.9,7.9)	5.1	(3.4,7.5)	4.1	(2.6,6.5)	-2.0	0.305	-13.9	0.027
Uganda	UGA	5.0	(3.9,6.4)	Decision making	4.0	(2.2,7.0)	5.1	(3.6,7.2)	5.2	(3.6,7.5)	1.2	0.582	-3.6	0.552
				Attitude towards violence	4.3	(2.7,6.7)	5.2	(3.3,8.2)	5.2	(3.9,7.1)	1.2	0.523	-2.8	0.641
				Social independence	2.7	(1.5,4.8)	1.6	(0.8,3.3)	0.9	(0.2,4.3)	-2.5	0.115	-19.0	0.083
Zambia	ZMB	1.8	(1.2,2.8)	Decision making	2.1	(0.8,5.1)	1.8	(0.8,4.0)	1.8	(1.0,3.2)	-0.1	0.924	0.8	0.941
				Attitude towards violence	3.1	(1.7,5.5)	1.4	(0.4,4.3)	1.2	(0.6,2.5)	-2.8	0.071	-18.8	0.156
				Social independence	20.1	(13.3,29.2)	9.5	(6.4,13.8)	7.1	(4.5,10.9)	-15.2	0.006	-23.9	0.001
Zimbabwe	ZWE	10.6	(8.1,13.7)	Decision making	31.7	(16.0,53.1)	13.4	(8.7,20.0)	8.5	(6.2,11.6)	-14.1	0.008	-21.1	0.001
				Attitude towards violence	12.5	(6.0,24.2)	12.1	(8.0,18.1)	9.4	(6.8,12.9)	-5.3	0.349	-9.3	0.246

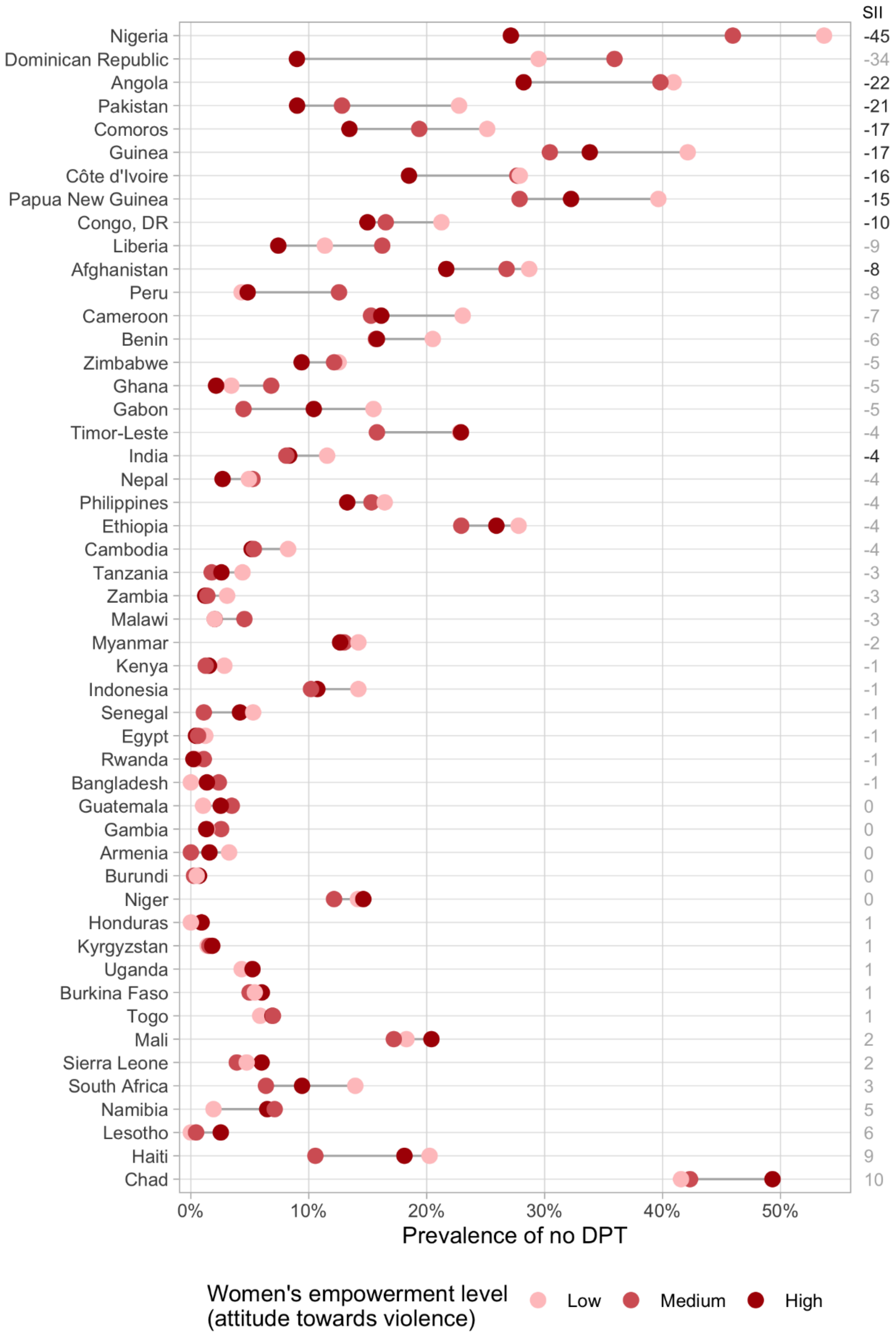

Figure S1 – No-DPT prevalence according to levels of SWPER's attitude towards violence domain by country. Light grey font indicates non-significant SII values.
Note: countries are ordered by the slope index of inequality (SII).
Estimates with N<25: Armenia(low), Dominican Republic (low and medium), Peru(low), Philippines(low), South Africa (low and medium).

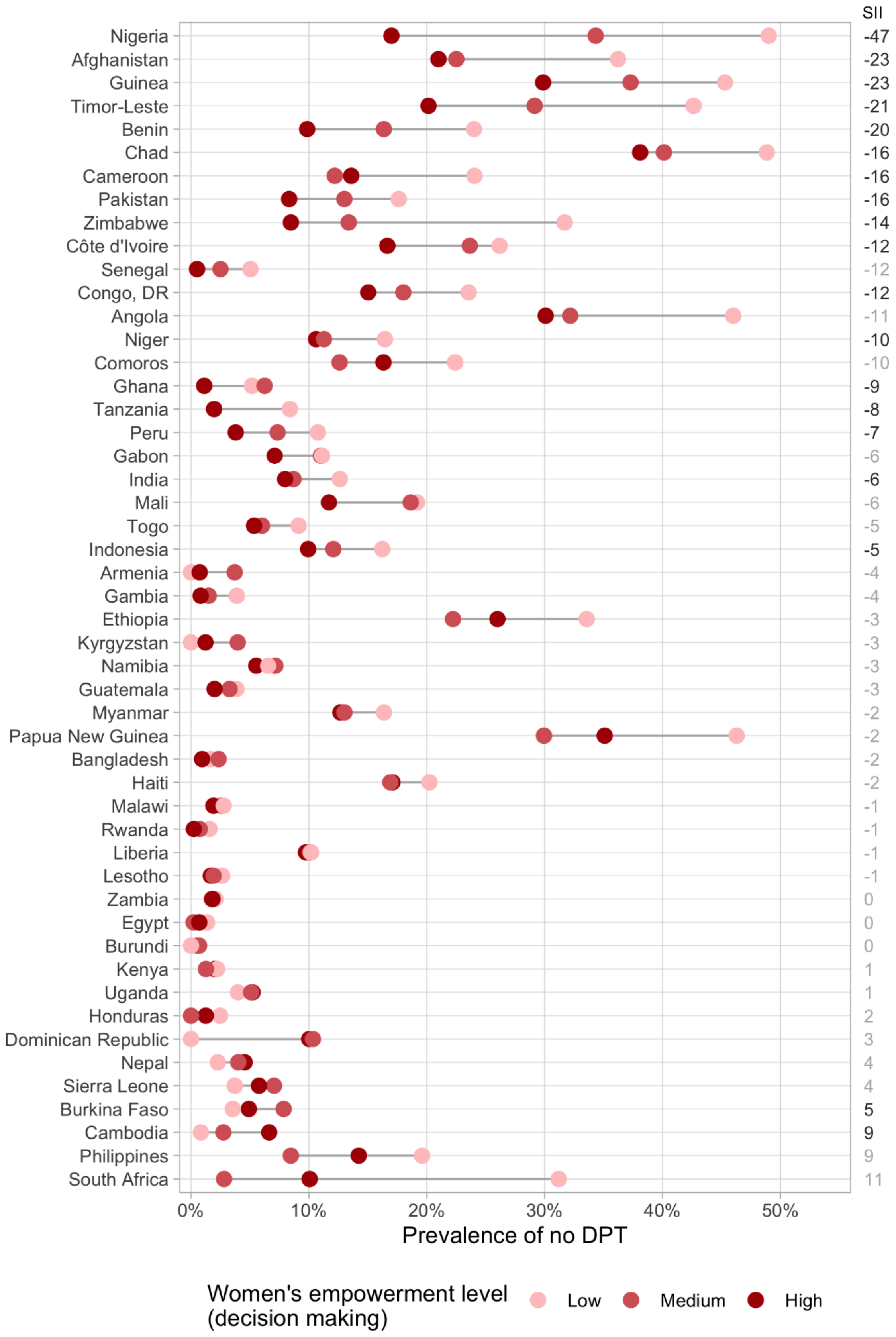

Figure S2 – No-DPT prevalence according to levels of SWPER's decision-making domain by country. Light grey font indicates non-significant SII values.
Note: countries are ordered by the slope index of inequality (SII). Estimates with N<25: Armenia(low), Cambodia(low), Dominican Republic (low), Lesotho(low), South Africa (low).

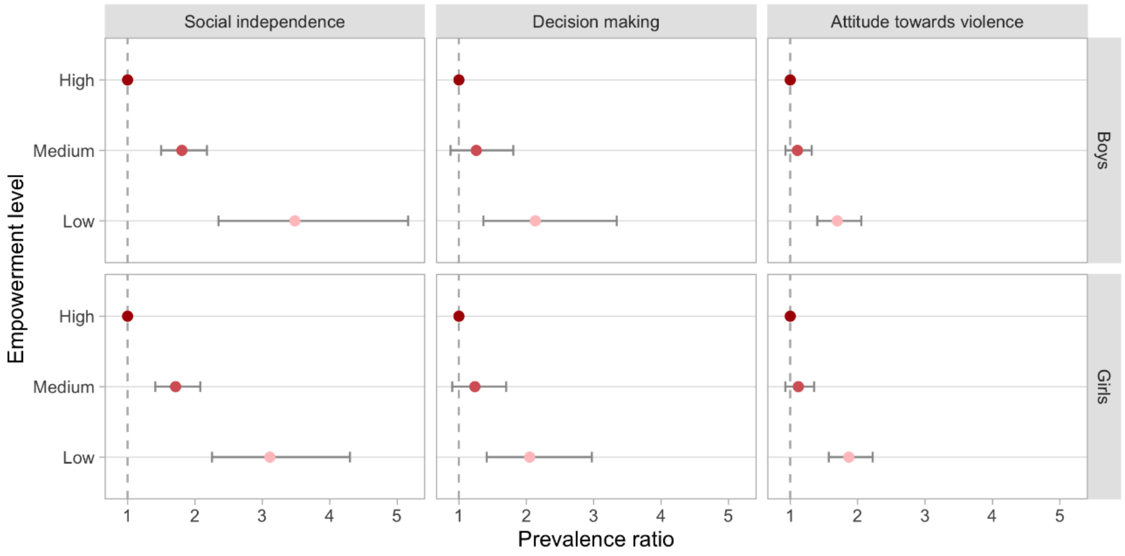

Figure S3 – Pooled prevalence ratio for no-DPT according to level of empowerment for each SWPER domain and by child's sex.

**Social independence domain**
Frequency of reading newspaper or magazine
Woman education in completed years of schooling
Age of woman at first birth
Age at first cohabitation
Age difference: woman's minus husband's age
Education difference: woman's minus husband's years of schooling
**Decision-making domain**
Who usually decides on respondent's health care
Who usually decides on large household purchases
Who usually decides on visits to family or relatives
**Attitude towards violence domain**
Beating justified if wife goes out without telling husband
Beating justified if wife neglects the children
Beating justified if wife argues with husband
Beating justified if wife refuses to have sex with husband
Beating justified if wife burns the food

Supp Table 2 : Items used in each domain of SWPER Global. Source: Ewerling, 2020.

Figure S4. Correlations between no-DPT prevalence and the proportion of women with a high level of empowerment for each SWPER domain, at country level excluding countries in the lowest no-DPT tercile.

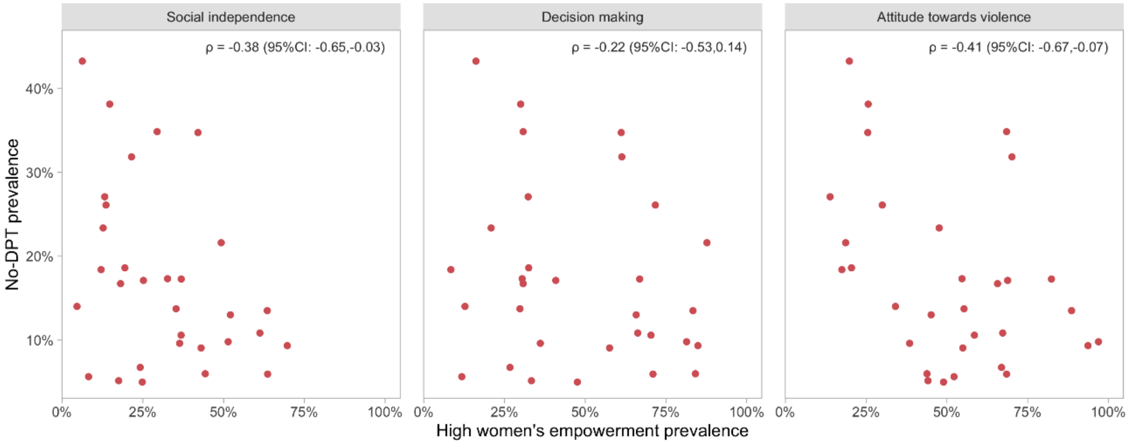

TS3: Pooled prevalence ratio for no-DPT according to level of empowerment for each SWPER domain adjusted for wealth quintiles.

	PR (95%CI)	PR (95%CI)	PR (95%CI)
	**Social independence**	**Decision making**	**Attitude towards violence**
**High**	1.00	1.00	1.00
**Medium**	1.4 (1.2; 1.7)	1.2 (0.9; 1.6)	1.0 (0.9; 1.2)
**Low**	2.5 (1.8; 3.4)	1.9 (1.3; 2.7)	1.5 (1.3; 1.8)
